# Supplementary material for: Lie prevalence, lie characteristics and strategies of self-reported good liars
Source: PLoS One. 2019 Dec 3;14(12):e0225566. doi: 10.1371/journal.pone.0225566 (PMC6890208; doi:10.1371/journal.pone.0225566)
Supplement: S1 File — (DOCX) [file pone.0225566.s001.docx]

**S1 File. Questionnaire definitions: Deception and strategies**

After providing informed consent, and prior to beginning the questionnaire, participants were provided the following set of definitions, which they were asked to read carefully and to keep in mind while making responses throughout the questionnaire: (1) “*To lie (i.e., deceive) is to successfully or unsuccessfully make a false statement (to another person), without forewarning, with the intention that the statement will be believed to be true (by the other person); misremembering is not the same as lying; a successful lie means that the intended false-belief has been produced, whereas an unsuccessful lie means that the intended false-belief has not been produced.”* (2) *“Strategies for telling lies (i.e., strategies of deception) refer to the self-regulatory method/s that an individual may use to increase their chances of telling a successful lie.”*

During the questionnaire, participants were asked to indicate how many lies, of several types, they had told within the past 24 hours. We described each of the options as follows: White lies (*Lies of this type occur when someone makes an inconsequential false statement with the purpose of easing social interactions*), Exaggerations (*Lies of this type occur when someone intentionally makes a statement that reports something as being better or worse than it really is*), Omissions/Concealment (*Lies of this type occur when someone intentionally withholds information, with the purpose of harming or disadvantaging the receiver for the liar’s own benefit*), Commission/ Fabrications (*Lies of this type occur when false information is intentionally presented as if it was true, with the purpose of harming or disadvantaging the receiver for the liar’s own benefit*), and Embedded lies (*Lies of this type occur when a statement contains one or more lies that are incorporated into an otherwise truthful story, for the liar’s own benefit*).

As well, during the questionnaire participants were asked to rate on a 10-point Likert scale (1 – not important to 10 – very important) how important they consider verbal and nonverbal strategies of deception to be for getting away with a lie. We described verbal strategies as: “*Relating to the control of an individual’s speech to say things that give a credible impression and avoid raising suspicion of possible deception”* and nonverbal strategies of deception as: *“Relating to the control of an individual’s behaviour to display body language that gives a credible impression and avoids raising suspicion of possible deception”.*
