# Supplementary material for: Lie prevalence, lie characteristics and strategies of self-reported good liars
Source: PLoS One. 2019 Dec 3;14(12):e0225566. doi: 10.1371/journal.pone.0225566 (PMC6890208; doi:10.1371/journal.pone.0225566)
Supplement: S2 File — (DOCX) [file pone.0225566.s002.docx]

**S2 File: Lie characteristics and distribution skewness**

Participants mostly reported telling white lies, and the distribution was non-normally distributed (skewness = 4.94, *SE* = 0.18; kurtosis = 31.57, *SE* = 0.35). Three participants mentioned telling over ten white lies in the past 24 hours, and their reports accounted for over 20% of the reported lies of this type. Forty-nine percent of participants reported having told no white lies in the same time frame. The distributions for the other types of lies (e.g., embedded lies, exaggerations) were similarly skewed in their distributions. Regarding the receivers of deception, participants reported lying most often to family members. Again, the distribution was non-normally distributed, with a skewness of 3.18 (*SE* = 0.18) and a kurtosis of 14.39 (*SE* = 0.35). Most participants (71.1%) reported not having told any lies to family members, whereas most of those who did lie to family (36.1%), told one or two lies. The highest reported number was eight lies told to family, which was only reported by one participant. The distributions for the other receivers of deception (e.g., friends, colleagues) were also skewed. Finally, participants reported telling most lies via face-to-face interactions (skewness = 3.17, *SE* = 0.18; kurtosis = 13.21, *SE* = 0.35). The majority (61.3%) did not report any lies during such interactions, whereas most participants who did report lies during face-to-face interactions (54.7%) reported only one or two lies. The distributions for the other mediums of deception (e.g., via text message or social media) were also non-normally distributed.
