# Supplementary material for: Lie prevalence, lie characteristics and strategies of self-reported good liars
Source: PLoS One. 2019 Dec 3;14(12):e0225566. doi: 10.1371/journal.pone.0225566 (PMC6890208; doi:10.1371/journal.pone.0225566)
Supplement: S3 File — (DOCX) [file pone.0225566.s003.docx]

**S3 File. Exploratory testing of liar characteristics**

Additionally, we explored the effects of sex and education level on self-reported deception ability. First, we evaluated the association between sex and self-reported deception ability by conducting a chi square test of association. In this analysis we used only males and females (95 and 97, respectively), we excluded two participants who responded ‘Prefer not to answer’ regarding their sex. We observed a significant association between sex and deception ability, χ^2^(2) = 12.31, *p* = .002, *V* = .253. We followed up this significant effect by comparing the column proportions. Our results indicate that, of those who self-reported to be Poor liars, 70% (*n* = 35) were female compared to 30% (*n* = 15) male. Additionally, of those who identified as Good liars, 62.7% (*n* = 42) were male whereas 37.3% (*n* = 25) were female. Both column proportions were significantly different at the .05 level. The distribution of males and females in the Neutral ability group did not differ significantly.

Next, to evaluate participants’ level of education, we classified ‘high school’, ‘some college’ and ‘associate degree’ as Low education (*n* = 71), ‘bachelor degree’ as Moderate education (*n* = 88), and ‘graduate or professional degree’ as High education (*n* = 35). To examine whether there was an association between education level and self-reported deception ability, we conducted a chi square test of association. There was no significant association between participants’ education level and their self-reported deception ability, χ^2^(4) = 9.09, *p* = .059, *V* = .153. Taken together, these findings suggest that men are more likely to self-report as Good liars, whereas women are more likely to self-report as Poor liars. Education level did not affect self-reported deception ability.
