# Supplementary material for: Lie prevalence, lie characteristics and strategies of self-reported good liars
Source: PLoS One. 2019 Dec 3;14(12):e0225566. doi: 10.1371/journal.pone.0225566 (PMC6890208; doi:10.1371/journal.pone.0225566)
Supplement: S4 File — (DOCX) [file pone.0225566.s004.docx]

**S4 File. Questionnaire part III: Recalling a serious lie**

Participants completed an additional section of the questionnaire that asked about a specific, personal instance in which they told a lie. Participants were asked to think back to a time when they told a serious lie and to answer the following questions accordingly. If they could not recall telling a serious lie, participants were given the option to answer the questions hypothetically. Before answering the questions, participants indicated whether they were responding based on an actual memory or regarding a hypothetical scenario. They were also asked to provide an open-ended description of the reason why they had lied. Afterwards, participants were asked to rate several items on 5-point Likert scales (1 – strongly agree to 5 – strongly disagree): (i) Before telling the lie, I prepared what I would say, (ii) I used one or more strategies when telling the lie, (iii) I was motivated to get away with the lie, and (iv) I was successful in getting away with the lie. Participants also responded to several open-ended questions: (i) Please describe why you did or did not prepare what you would say during your lie, (ii) If you did prepare before telling your lie, how did you prepare? (iii) Please explain the strategy or strategies you used to be convincing with this lie, and (iv) Please describe why you think you were or were not successful in getting away with your lie.

**Reliability**. The main coder and a second coder coded a randomly selected 20% of the participants’ open-ended responses in order to establish inter-rater reliability regarding the classification of responses into the appropriate categories. This was evaluated using the two-way mixed effects model measuring consistency, which indicated that raters were consistent across all question categories. Specifically, the Single Measures *ICCs* for question one (“What was the reason that you lied?”) ranged from .80 to 1.00; for question two (“Please describe why you did or did not prepare what you would say during your lie?”) ranged from .53 to .85; for question three (“If you did prepare, how did you prepare?”) ranged from .65 to .88; for question four (“What strategy or strategies did you use to be convincing with this lie?”) ranged from .55 to 1.00; and for question five (“Please describe why you think you were or were not successful in getting away with your lie?”) ranged from .53 to .85. After confirming that the raters were consistent, the main coder (first author) completed the remaining sample of participant responses and only these scores were used in the analysis.

**Deceivers’ strategies as a function of rationale**

We were interested in exploring how people strategically deceive, as a function of their rationale for deceiving. In the final section of the questionnaire, participants were asked to recall a time in which they had told a serious lie. Based on their responses to this question, we created five categories of rationales for deceiving, as follows: (i) for personal gain (*n* = 41), (ii) to avoid consequence (*n* = 47), (iii) to avoid the situation (*n* = 34), (iv) to benefit another person (*n* = 22), and (v) other/miscellaneous (*n* = 11). We included only those participants who reported to be basing their responses on a real memory, rather than a hypothetical scenario. This led to the exclusion of 39 participants, and thus, our analyses for the current section were conducted on 155 participants.

Overall, participants reported having been motivated to get away with their lie (*M* = 1.68, *SD* = 0.95), and there were no statistically significant differences between the five rationale groups, *F*(4, 150) = .38, *p* = .824, η_P_^2^ = .010; *BF_01_* = 24.39. Participants also reported to have prepared what they would say prior to telling the lie (*M* = 1.98, *SD* = 1.14); however, there were no differences between the five rationale groups, *F*(4, 150) = .07, *p* = .991, η_P_^2^ = 002; *BF_01_* = 36.31.

**Strategies.** Participants reported using one or more strategies when telling their lie (*M* = 2.12, *SD* = 1.06); however, there were no differences between the five rationale groups in endorsing at least one strategy, *F*(4, 190) = 1.24, *p* = .296, η_P_^2^ = .032; *BF_01_* = 6.90. Supplementary Table 1 provides an overview of the strategy categories that emerged from the qualitative coding of participants’ responses to the question regarding the strategies they employed when delivering their lie. Overall, participants across groups endorsed the various strategies a total of 228 times. Those who deceived to avoid a negative consequence or for personal gain reported the most strategies (a total of 57 and 56 strategies, respectively), whereas those who lied for miscellaneous reasons reported the fewest strategies (11 mentioned strategies; see Supplementary Table 1). The most frequently endorsed strategies across groups were *“Behavioral manipulation”* (22.37% of all reported strategies; e.g., maintaining eye contact, having a steady voice, appearing calm, behaving in the same manner as normal, etcetera) and *“The inclusion of certain types of information”* (17.98% of all reported strategies; e.g., providing unverifiable or verifiable details, including timestamps and specific descriptions, etcetera).

| **Supplementary Table 1. Qualitative strategies for telling a serious lie as a function of rationale** | | | |
| --- | --- | --- | --- |
| Strategy Categories: | *N* | *M* | *SD* |
| Behavioral manipulation | 51 | 0.33 | 0.47 |
| For personal gain | 16 | 0.39 | 0.49 |
| To avoid consequence | 19 | 0.40 | 0.49 |
| To avoid the situation | 9 | 0.26 | 0.45 |
| To benefit another person | 6 | 0.27 | 0.46 |
| Miscellaneous | 1 | 0.10 | 0.32 |
| Including certain types of information | 41 | 0.26 | 0.44 |
| For personal gain | 8 | 0.20 | 0.40 |
| To avoid consequence | 14 | 0.29 | 0.46 |
| To avoid the situation | 14 | 0.42 | 0.50 |
| To benefit another person | 4 | 0.18 | 0.40 |
| Miscellaneous | 1 | 0.10 | 0.32 |
| General strategies | 28 | 0.18 | 0.39 |
| For personal gain | 8 | 0.20 | 0.40 |
| To avoid consequence | 7 | 0.15 | 0.36 |
| To avoid the situation | 5 | 0.15 | 0.36 |
| To benefit another person | 5 | 0.23 | 0.43 |
| Miscellaneous | 3 | 0.30 | 0.48 |
| Keeping the story short and simple | 27 | 0.17 | 0.38 |
| For personal gain | 6 | 0.15 | 0.36 |
| To avoid consequence | 8 | 0.17 | 0.38 |
| To avoid the situation | 8 | 0.24 | 0.43 |
| To benefit another person | 3 | 0.14 | 0.35 |
| Miscellaneous | 2 | 0.20 | 0.42 |
| Telling a plausible and convincing story | 18 | 0.12 | 0.32 |
| For personal gain | 6 | 0.15 | 0.36 |
| To avoid consequence | 7 | 0.16 | 0.36 |
| To avoid the situation | 2 | 0.06 | 0.24 |
| To benefit another person | 2 | 0.09 | 0.29 |
| Miscellaneous | 1 | 0.10 | 0.32 |
| Incorporating truthful details | 21 | 0.14 | 0.34 |
| For personal gain | 10 | 0.24 | 0.43 |
| To avoid consequence | 6 | 0.13 | 0.33 |
| To avoid the situation | 2 | 0.06 | 0.24 |
| To benefit another person | 3 | 0.14 | 0.35 |
| Miscellaneous | 0 | 0.00 | 0.00 |
| Omitting certain types of information | 20 | 0.13 | 0.34 |
| For personal gain | 6 | 0.15 | 0.36 |
| To avoid consequence | 3 | 0.06 | 0.24 |
| To avoid the situation | 5 | 0.15 | 0.36 |
| To benefit another person | 5 | 0.23 | 0.43 |
| Miscellaneous | 1 | 0.10 | 0.32 |
| Emotional manipulation | 12 | 0.08 | 0.27 |
| For personal gain | 2 | 0.05 | 0.22 |
| To avoid consequence | 6 | 0.13 | 0.33 |
| To avoid the situation | 2 | 0.06 | 0.24 |
| To benefit another person | 2 | 0.09 | 0.29 |
| Miscellaneous | 0 | 0.00 | 0.00 |
| Not Applicable | 6 | 0.04 | 0.19 |
| For personal gain | 1 | 0.02 | 0.16 |
| To avoid consequence | 1 | 0.02 | 0.14 |
| To avoid the situation | 2 | 0.06 | 0.24 |
| To benefit another person | 0 | 0.00 | 0.00 |
| Miscellaneous | 2 | 0.20 | 0.42 |
| No Strategy | 4 | 0.03 | 0.16 |
| For personal gain | 1 | 0.02 | 0.16 |
| To avoid consequence | 0 | 0.00 | 0.00 |
| To avoid the situation | 1 | 0.03 | 0.17 |
| To benefit another person | 1 | 0.05 | 0.21 |
| Miscellaneous | 1 | 0.10 | 0.32 |
| *Note.* The *N* column represents the number of participants who endorsed each category, both in the total sample and for each rationale group, respectively. The total number of endorsed categories surpasses 155 because each participant could report multiple strategies that may have fallen into more than one category. | | | |

**The influence of lie rationale on participants’ preparation**

To further evaluate whether participants prepared more or less depending on their rationale for lying, we created two groups to represent “preparers” (scores of one and two) and “non-preparers” (scores of four and five) based on participants’ responses to their preparation on a 5-point Likert scale. The majority of participants reported to have prepared (*n* = 125) whereas only 24 participants indicated that they did not prepare. Six participants responded in a neutral manner (i.e., scores of three) and thus their data were removed from the analyses. To examine whether there was an association between whether an individual prepares for their deception or not and the rationale for deceiving, we conducted a series of chi square tests of independence. All expected cell frequencies were greater than five. There was not a statistically significant association between preparation and the rationale for deceiving, *χ^2^*(4) = 0.72, *p* = .948, *V* = .070.

Supplementary Table 2 provides an overview of the data regarding participants’ coded responses to the open-ended question: “Please describe why you did or did not prepare what you would say during your lie.” These data are reported on only the preparer and non-preparer groups. The majority of justifications fell into the categories relating to preparing for the deception; whereas almost a quarter of the reports (21.12%) fell into the two categories relating to a lack of preparation (i.e., *“No preparation because caught unexpectedly”* and *“Miscellaneous reasons for not preparing”*). Across groups, the most endorsed reason for preparing was to *“Ensure the lie is sound and convincing”* (17.39% of reports). The two rationales that led to the highest endorsement of preparation methods were those who told lies to avoid consequence and for personal gain (mentioned 50 and 43 justifications, respectively).

| **Supplementary Table 2. Justifications for preparing or not preparing as a function of rationale** | | | |
| --- | --- | --- | --- |
| Justifications: | *N* | *M* | *SD* |
| Prepare to ensure the lie is sound and convincing | 28 | 0.19 | 0.39 |
| For personal gain | 5 | 0.12 | 0.33 |
| To avoid consequence | 9 | 0.19 | 0.40 |
| To avoid the situation | 10 | 0.32 | 0.48 |
| To benefit another person | 3 | 0.15 | 0.37 |
| Miscellaneous | 1 | 0.10 | 0.32 |
| Prepare to increase the likelihood of getting away with the lie | 23 | 0.15 | 0.36 |
| For personal gain | 3 | 0.07 | 0.26 |
| To avoid consequence | 10 | 0.21 | 0.41 |
| To avoid the situation | 7 | 0.23 | 0.43 |
| To benefit another person | 2 | 0.10 | 0.31 |
| Miscellaneous | 1 | 0.10 | 0.32 |
| Miscellaneous reasons for preparing | 25 | 0.17 | 0.38 |
| For personal gain | 7 | 0.17 | 0.38 |
| To avoid consequence | 7 | 0.15 | 0.36 |
| To avoid the situation | 5 | 0.16 | 0.37 |
| To benefit another person | 4 | 0.20 | 0.41 |
| Miscellaneous | 2 | 0.20 | 0.40 |
| Preparing to control information disclose | 21 | 0.14 | 0.35 |
| For personal gain | 8 | 0.20 | 0.40 |
| To avoid consequence | 6 | 0.13 | 0.34 |
| To avoid the situation | 4 | 0.13 | 0.34 |
| To benefit another person | 3 | 0.15 | 0.37 |
| Miscellaneous | 0 | 0.00 | 0.00 |
| No preparation because caught unexpectedly | 20 | 0.13 | 0.34 |
| For personal gain | 4 | 0.10 | 0.30 |
| To avoid consequence | 8 | 0.17 | 0.38 |
| To avoid the situation | 0 | 0.00 | 0.00 |
| To benefit another person | 6 | 0.30 | 0.47 |
| Miscellaneous | 2 | 0.20 | 0.42 |
| Miscellaneous reasons for not preparing | 14 | 0.09 | 0.29 |
| For personal gain | 5 | 0.12 | 0.33 |
| To avoid consequence | 2 | 0.04 | 0.20 |
| To avoid the situation | 2 | 0.06 | 0.25 |
| To benefit another person | 4 | 0.20 | 0.41 |
| Miscellaneous | 1 | 0.10 | 0.32 |
| Not Applicable | 30 | 0.20 | 0.40 |
| For personal gain | 11 | 0.27 | 0.45 |
| To avoid consequence | 8 | 0.17 | 0.38 |
| To avoid the situation | 7 | 0.23 | 0.43 |
| To benefit another person | 1 | 0.05 | 0.22 |
| Miscellaneous | 3 | 0.30 | 0.48 |
| *Note.* The *N* column represents the number of participants who endorsed each category, both in the total sample and for each rationale group, respectively. The total number of endorsed categories surpasses 149 because each participant could report multiple strategies that may have fallen into more than one category. We coded for an additional category, “Intentionally no preparation to utilize spontaneity”; however, of the data retained in the analyses, there was no endorsement of this category. | | | |

Supplementary Table 3 provides an overview of the methods of preparation that arouse from the qualitative coding of participants’ responses. Two techniques were reported most often by participants: *“Carefully planning a plausible story”* (26.49% of the total reported techniques fell into this category) and *“Rehearsing the story”* (22.70% of the total reported techniques). Participants who reported to be deceiving to avoid consequences or for personal gain endorsed the highest frequency of preparation techniques (55 and 52 mentioned techniques, respectively).

| **Supplementary Table 3. Preparation techniques as a function of rationale** | | | |
| --- | --- | --- | --- |
| Preparation Techniques: | *N* | *M* | *SD* |
| Carefully plan a plausible story | 49 | 0.32 | 0.47 |
| For personal gain | 12 | 0.29 | 0.46 |
| To avoid consequence | 15 | 0.31 | 0.47 |
| To avoid the situation | 14 | 0.42 | 0.50 |
| To benefit another person | 6 | 0.27 | 0.46 |
| Miscellaneous | 2 | 0.20 | 0.42 |
| Rehearse the story | 42 | 0.27 | 0.45 |
| For personal gain | 9 | 0.22 | 0.42 |
| To avoid consequence | 12 | 0.25 | 0.44 |
| To avoid the situation | 14 | 0.42 | 0.50 |
| To benefit another person | 7 | 0.32 | 0.48 |
| Miscellaneous | 0 | 0.00 | 0.00 |
| Use imagination | 26 | 0.17 | 0.38 |
| For personal gain | 10 | 0.24 | 0.44 |
| To avoid consequence | 7 | 0.15 | 0.36 |
| To avoid the situation | 3 | 0.09 | 0.29 |
| To benefit another person | 5 | 0.23 | 0.43 |
| Miscellaneous | 1 | 0.10 | 0.32 |
| Not Applicable | 24 | 0.15 | 0.36 |
| For personal gain | 7 | 0.17 | 0.38 |
| To avoid consequence | 7 | 0.15 | 0.36 |
| To avoid the situation | 3 | 0.09 | 0.29 |
| To benefit another person | 4 | 0.18 | 0.39 |
| Miscellaneous | 3 | 0.30 | 0.48 |
| No preparation | 19 | 0.12 | 0.33 |
| For personal gain | 4 | 0.10 | 0.30 |
| To avoid consequence | 5 | 0.10 | 0.31 |
| To avoid the situation | 3 | 0.09 | 0.29 |
| To benefit another person | 4 | 0.18 | 0.39 |
| Miscellaneous | 3 | 0.30 | 0.48 |
| Gather certain information to include | 14 | 0.09 | 0.29 |
| For personal gain | 3 | 0.07 | 0.26 |
| To avoid consequence | 7 | 0.15 | 0.36 |
| To avoid the situation | 4 | 0.12 | 0.33 |
| To benefit another person | 0 | 0.00 | 0.00 |
| Miscellaneous | 0 | 0.00 | 0.00 |
| Plan the nonverbal behavior | 11 | 0.07 | 0.26 |
| For personal gain | 7 | 0.17 | 0.38 |
| To avoid consequence | 2 | 0.04 | 0.20 |
| To avoid the situation | 1 | 0.03 | 0.17 |
| To benefit another person | 0 | 0.00 | 0.00 |
| Miscellaneous | 1 | 0.10 | 0.32 |
| *Note.* The *N* column represents the number of participants who endorsed each category, both in the total sample and for each rationale group, respectively. The total number of endorsed categories surpasses 155 because each participant could report multiple strategies that may have fallen into more than one category. | | | |

**Perceived success of a serious lie**

Lastly, participants reported to have been successful in getting away with their lie (*M* = 1.84, *SD* = 1.06); however, there were no differences between the five rationale groups, *F*(4, 150) = .60, *p* = .663, η_P_^2^ = .016; *BF_01_* = 15.86. We also examined participants’ qualitative responses to the question “Please describe why you think you were or were not successful in getting away with your lie.” Supplementary Table 4 provides an overview of the exact values of participants’ reasons for believing their deception was successful versus unsuccessful, as a function of their rationale. The qualitative data mirrored participants’ Likert response scores; participants most frequently endorsed the category that indicated they thought to have been successful because the receiver believed the lie (34.94% of the reports; e.g., the receiver did not question further, the receiver’s behavior/response indicated acceptance of the story, the receiver was not suspicious, etcetera).

| **Supplementary Table 4. Reasons related to successful versus unsuccessful deception as a function of rationale** | | | |
| --- | --- | --- | --- |
| Reasons relating to perceived success: | *N* | *M* | *SD* |
| Successful: Receiver believed the lie | 58 | 0.37 | 0.49 |
| For personal gain | 17 | 0.42 | 0.50 |
| To avoid consequence | 17 | 0.35 | 0.48 |
| To avoid the situation | 14 | 0.41 | 0.50 |
| To benefit another person | 8 | 0.36 | 0.49 |
| Miscellaneous | 2 | 0.20 | 0.42 |
| Successful: Strategic statement and delivery | 43 | 0.28 | 0.45 |
| For personal gain | 13 | 0.32 | 0.47 |
| To avoid consequence | 15 | 0.31 | 0.47 |
| To avoid the situation | 8 | 0.24 | 0.43 |
| To benefit another person | 5 | 0.23 | 0.43 |
| Miscellaneous | 2 | 0.20 | 0.42 |
| Successful: No repercussions | 20 | 0.13 | 0.34 |
| For personal gain | 4 | 0.10 | 0.30 |
| To avoid consequence | 12 | 0.25 | 0.44 |
| To avoid the situation | 3 | 0.09 | 0.29 |
| To benefit another person | 1 | 0.05 | 0.21 |
| Miscellaneous | 0 | 0.00 | 0.00 |
| Not successful: General | 17 | 0.11 | 0.31 |
| For personal gain | 4 | 0.10 | 0.30 |
| To avoid consequence | 2 | 0.04 | 0.20 |
| To avoid the situation | 4 | 0.12 | 0.33 |
| To benefit another person | 4 | 0.18 | 0.39 |
| Miscellaneous | 3 | 0.30 | 0.48 |
| Neutral: Unaware of deception success | 19 | 0.12 | 0.33 |
| For personal gain | 3 | 0.07 | 0.26 |
| To avoid consequence | 8 | 0.17 | 0.38 |
| To avoid the situation | 3 | 0.09 | 0.29 |
| To benefit another person | 5 | 0.23 | 0.43 |
| Miscellaneous | 0 | 0.00 | 0.00 |
| Not Applicable | 9 | 0.06 | 0.24 |
| For personal gain | 2 | 0.05 | 0.22 |
| To avoid consequence | 1 | 0.02 | 0.14 |
| To avoid the situation | 2 | 0.06 | 0.24 |
| To benefit another person | 1 | 0.05 | 0.21 |
| Miscellaneous | 3 | 0.30 | 0.48 |
| *Note.* The *N* column represents the number of participants who endorsed each category, both in the total sample and for each rationale group, respectively. The total number of endorsed categories surpasses 155 because each participant could report multiple strategies that may have fallen into more than one category. | | | |
