# Supplementary material for: Lie prevalence, lie characteristics and strategies of self-reported good liars
Source: PLoS One. 2019 Dec 3;14(12):e0225566. doi: 10.1371/journal.pone.0225566 (PMC6890208; doi:10.1371/journal.pone.0225566)
Supplement: Attachment — Submitted filename: Response to Reviewers.docx [file pone.0225566.s005.docx]

To the editor, PLOS ONE

Dear Professor Sartori,

We thank you and the reviewers for your constructive feedback regarding our manuscript entitled “Lie prevalence, lie characteristics and strategies of self-reported good liars” (Manuscript Number PONE-D-19-22731), and for the opportunity to address these comments. Below, we itemize in italics how we dealt with each of the comments. For your and the reviewers’ convenience, we used the track changes version of word to highlight the changes we made.

**Response to Reviewer #1:**

1. Concerning the data collection procedure, the authors seem aware of the limitations of using crowd-sourcing platforms in scientific research and the strategies and constraints employed seem enough for ensuring the data quality. Nonetheless, as stated by the authors, the inconsistencies emerged between the response to the qualitative strategy question and the multiple-response strategy question could be due to inattentive or careless responding by participants (a common issue when collecting data through crowd-sourcing platforms such as mTurk). In order to exclude this effect, a possible solution could be to collect an additional small sample in a more controlled setting (i.e. in the laboratory) and comparing the new results with the ones reported in this version of the manuscript.

*Indeed, we expressed concerns about the inconsistency in our data between the qualitative and multiple-response strategy questions. Although we originally proposed careless responding as a potential explanation for this inconsistency, during our work on the revision, we realized that a more theoretically based explanation lies in the measurement procedures. Specifically, we examined liars’ strategies in two ways: i) using an inductive data-driven approach and ii) using a deductive theory-driven approach. The data-driven approach involved the construction of a coding scheme based on participants’ open-ended responses about their strategy use. Several limitations exist regarding this type of data. First, it is difficult to articulate the reasons for one’s behaviour in retrospect, particularly for a complex phenomenon such as deception, which means we may have coded incomplete or inaccurate responses (Ericsson & Simon, 1980). Second, although we ensured high consistency between coders (ICCs between .79 and 1.00), data-driven approaches are inherently susceptible to human subjectivity, error, and bias (e.g., Morrissey, 1974; Syed & Nelson, 2015). Third, given the breadth of strategy responses that we observed at the individual level, our coding process required the reduction of responses into a practical number of broad categories that could be submitted to statistical procedures to produce meaningful results. The data-driven approach therefore led to the elimination of more nuanced and informative units of data. In contrast, the theory-driven approach involved gathering existing psychological theory regarding deceivers’ strategies and cues to deception and compiling a predefined list of potential strategies for participants to select from. This type of research has been used extensively in the deception literature; for instance, researchers have historically preferred to use this closed question method for measuring cues to deception whereby participants are provided with a list of verbal and behavioural cues which they can indicate their belief about (e.g., Vrij, 2008). Moreover, these data may be superior given that they are less susceptible to the above-mentioned limitations that hinder data-driven coding. There is also empirical evidence to suggest that open- and closed-ended responses involve different memory (Yonelinas, 2002) or cognitive processes entirely (Frew, Wolstenholme, & Whynes, 2004). In sum, the differences associated with the data- versus theory-driven research procedures may account for the inconsistency in our results regarding liars’ strategies. We have now clarified our views on this issue on Pg. 18.*

*Our study involved a substantial number of statistical comparisons (i.e., 18 correlational analyses, 7 chi-square tests of independence, and 12 between-subjects ANOVAs)^[1]^. To determine the sample size required to validate our data in a second study, we conducted several power analyses for each statistical parameter in the study^[2]^. We report here the greatest obtained sample size: A priori power analysis suggested that to achieve an 85% likelihood of detecting a true difference given a medium effect size (.25; Cohen, 1988), 180 participants would be required, for a one-way between-subjects ANOVA. Collecting a sample of this size in the laboratory was unfortunately not feasible due to resource constraints. The alternative, as suggested by the reviewer, would be to collect an additional small sample (e.g., 25% of our original sample; n = 50). Post hoc power analysis revealed that the statistical power for a sample size of 50 would have been less than adequate (power of .32) to detect a medium effect size (.25), for a one-way ANOVA. We believe that comparing the results from a low-powered sample to our original results would be unreliable. We have now added to our Discussion section that future research should attempt to directly replicate our survey with different populations, for instance, students tested in the laboratory. As well, we now suggest that conceptual replication would be an interesting avenue of future research to evaluate how our findings fare against different methodological approaches to measuring deception ability and good liars’ strategies. See Pg. 20*

*All in all, we believe that the advantages of using mTurk data for the purposes of this exploratory study outweigh the disadvantages. This platform allowed us to access a population that is arguably more representative of criminal liars compared to undergraduate-student samples typically used in deception studies. Moreover, previous research has shown that data produced via mTurk is of sufficient quality. That is, samples collected on mTurk are no better or worse than traditional convenience samples from university students (Fleischer, Mead, & Huang, 2015; Landers & Behrend, 2015), and surveys conducted on mTurk can produce a representative sample of the United States population that yields results akin to those observed from more expensive survey techniques (Simons & Chabris, 2012).*

2. Among the described findings, the self-reported good liars seem mostly telling inconsequential lies (white lies) and placing a higher value on verbal strategies than on behavioural ones for successfully deceiving. However, the results described in the supplementary materials concerning the last section of the questionnaire (where participants were asked which kind of strategies they use when telling serious lies) highlighted that "Behavioural manipulation" was the most frequently endorsed strategy. The authors are asked to explain this contrasting result.

*Our main analyses indeed revealed that self-reported good liars rated verbal strategies as significantly more important than neural or poor liars; however, across deception ability groups, all participants expressed behavioural strategies as being important for deceiving successfully. As such, it is not entirely contradictory that our supplemental analyses showed that all liars relied on strategies relating to behavioural manipulation when telling lies. Moreover, although behavioural manipulation was the most frequently mentioned strategy for telling lies of serious consequence (in the last section of the questionnaire), this strategy represented less than a quarter (22%) of all reported strategies. Endorsed nearly as frequently was the strategy of including certain types of verbal information (18% of all reported strategies). Thus, the results of this supplemental analysis do not imply that liars disproportionately rely on behavioural strategies when telling lies of serious consequence.*

*We have now further inspected the final section of the questionnaire. Specifically, we checked the strategy endorsement across deception ability groups, finding that – for Good, Neutral, and Poor liars alike – the two most frequently reported strategies were ‘Behavioural manipulation’ and ‘Including certain types of information’. Interestingly, in their reports regarding strategy use while telling a serious lie, Good liars’ third most reported strategy was ‘Incorporating truthful information’ (reported by 14 participants), whereas Poor and Neutral liars reported this strategy infrequently (reported by 4 and 3 participants, respectively). The third most frequently endorsed strategy for Poor liars was to ‘Tell a plausible story’ (7 participants), and for Neutral liars it was to ‘Keep the story short and simple’ (14 participants). Taken together, these findings largely adhere to the patterns reported in the main components of the questionnaire. Given this overlap, and to conserve manuscript length, we opted not to include these additional analyses in the manuscript. An interesting avenue for future research could be to explore how good liars’ strategies fluctuate as a factor of different deception rationales.*

**Response to Reviewer #2:**

1. To encourage open science and data reproducibility, the authors should publish their data in a repository, so the link to the data should be made available and open.

*We made all data underlying the findings of our manuscript available on Open Science Framework (OSF). We provided an anonymous view-only link on the PLOS ONE manuscript submission platform. Upon publication of this article, the link to these datasets will be publicly available on OSF.*

2. References: I noticed that the number of self-citations is very high. 11 out of 53 references (20%) contain at least one of the authors of the present paper. I invite the authors to reduce self-references.

*We have reduced the number of self-citations to 9 out of 63 total references (14%).*

3. Data are collected through Amazon Mechanical Turk. To addressed the limitations commonly associated with this type of study (these limitations are mentioned by the authors in the manuscript as well, see also <http://www.annualreviews.org/doi/abs/10.1146/annurev-clinpsy-021815-093623> and <http://journals.plos.org/plosone/article?id=10.1371/journal.pone.0057410#s15>), I suggest to expand the sample with additional subjects collected in laboratory. This addition sample can be used to confirm the results obtained from the Turkers.

*We have now added these references to the manuscript (Pgs. 19-20). Regarding data validation, please refer to our response to reviewer #1, comment #1.*

4. Participants: information about schooling is missed. I think that this is a variable that should not be underestimated. I would like to see a discussion about how this variable may influence the self-reported perception about the ability to lie and the strategy used. If the authors collected this data, I’d like to see some statistics comparing high and low schooling participants and males vs females. For example, are self-reported good liars mostly males with high educational level?

*The largest portion of participants reported having a Bachelor’s degree (45.4% of the sample), followed by some college (20.1%), a Master’s degree (16.5%), an Associate degree (11.9%), a high school diploma (4.6%), a Professional degree (1%), or a Doctoral degree (0.5%). We have now included a description regarding the association between self-reported deception ability and participants’ education level and sex, as follows:*

*“Finally, we also explored the associations between sex and education level on laypeople’s self-reported deception ability by conducting a series of chi square tests of association. We observed a significant association between sex and deception ability, χ2(2) = 12.31, p = .002, V = .253. Further examination revealed that, of those who self-reported to be Poor liars, 70% (n = 35) were female compared to 30% (n = 15) male. Additionally, of those who identified as Good liars, 62.7% (n = 42) were male whereas 37.3% (n = 25) were female. Both column proportions were significantly different at the .05 level. We did not observe a significant association between participants’ education level and their self-reported deception ability, χ2(4) = 9.09, p = .059, V = .153. The complete analyses are presented in the Supporting Information.” Pg. 15*

5. Which are the practical implications of this study? How this study enriches the state of the art? And how this study can concretely influence the development of more efficient lie detection machines? Please, discuss this point.

*We now include a paragraph outlining the implications of this research and we address ideas for future avenues of investigation, as follows:*

*“This study explored the deception characteristics and strategies used by self-reported good liars. Deception researchers generally agree that the most diagnostic information is found in the content of liars’ speech [59]. Content-based cues to deception, however, may be less effective for detecting good liars who rely highly on verbal strategies of deception. This could even offer an explanation for the modest effect sizes observed in the deception literature [60]. For instance, good liars in our study reported to strategically embed their lies into truthful information. This finding has potential implications for the reliability of credibility assessment tools that derive from the assumption that truth tellers’ statements are drawn from memory traces whereas liars’ statements are fabricated from imagination [61, 62]. If good liars draw on their memory of truthful previous experiences, then their statements may closely resemble those of their truth telling counterparts. Another interesting observation was that self-reported good liars were more likely than poor liars to provide unverifiable details. This fits with the findings of previous literature on the VA, which contends that liars provide information that cannot be verified to balance their goals of being perceived as cooperative and of minimizing the chances of falsification by investigators [32, 33]. A fruitful avenue of future research could be to further explore liars’ strategic inclusion of truthful information and unverifiable details. Doing so may give lie detectors an advantage for unmasking skilled liars. It would also be interesting for future research to examine how good versus poor liars are affected by certain interview techniques designed to increase the difficulty of lying such as the reverse-order technique [63].” Pg. 20*

**Additional Manuscript Changes:**

We made minor changes to sentence structure throughout the manuscript.

**References:**

Bond, J. C. F., & DePaulo, B. M. (2006). Accuracy of deception judgments. Personality and Social Psychology Review, 10, 214–234. doi:10.1207/s15327957pspr1003_2

Chandler, J., & Shapiro, D. (2016). Conducting clinical research using crowdsourced convenience samples. Annual review of clinical psychology, 12.

Cohen, J. (1988). Statistical power analysis for the behavioral sciences (2nd ed.). Hillsdale, N.J: L. Erlbaum Associates.

Crump, M. J., McDonnell, J. V., & Gureckis, T. M. (2013). Evaluating Amazon's Mechanical Turk as a tool for experimental behavioral research. PloS one, 8, e57410.

Evans, J. R., Michael, S. W., Meissner, C. A., & Brandon, S. E. (2013). Validating a new assessment method for deception detection: Introducing a Psychologically Based Credibility Assessment Tool. *Journal of Applied Research in Memory and Cognition, 2,* 33-41.

Fleischer, A., Mead, A. D., & Huang, J. (2015). Inattentive responding in MTurk and other online samples. *Industrial and Organizational Psychology, 8,* 196-202. doi: 10.1017/iop.2015.25

Frew, E. J., Wolstenholme, J. L., & Whynes, D. K. (2004). Comparing willingness-to-pay: bidding game format versus open-ended and payment scale formats. Health Policy, 68(3), 289-298.

Johnson, M. K., & Raye, C. L. (1981). Reality monitoring. Psychological Review, 88(1), 67-85. doi: 10.1037/0033-295X.88.1.67

Luke, T. J. (2019). Lessons from Pinocchio: Cues to deception may be highly exaggerated. Perspectives on Psychological Science, 14, 646-671.

Morrissey, E. R. (1974). Sources of error in the coding of questionnaire data. Sociological Methods & Research, 3(2), 209-232.

Simons DJ, Chabris CF. Common (mis) beliefs about memory: A replication and comparison of telephone and Mechanical Turk survey methods. PloS one. 2012 Dec 18;7(12):e51876.

Steller, M., & Köhnken, G. (1989). Criteria-based content analysis. In Raskin, D. C. (Ed.), Psychological methods in criminal investigation and evidence (pp. 217–245). New York: Springer Publishing Company.

Syed, M., & Nelson, S. C. (2015). Guidelines for establishing reliability when coding narrative data. Emerging Adulthood, 3(6), 375-387.

Wright, G. R., Berry, C. J., & Bird, G. (2012). “You can't kid a kidder”: association between production and detection of deception in an interactive deception task. *Frontiers in human neuroscience, 6,* 87.

Wright, G. R., Berry, C. J., & Bird, G. (2013). Deceptively simple… The “deception-general” ability and the need to put the liar under the spotlight. *Frontiers in neuroscience, 7,* 152.

Yonelinas, A. P. (2002). The nature of recollection and familiarity: A review of 30 years of research. Journal of memory and language, 46(3), 441-517.

1. These numbers do not include the supplementary analyses reported in the Supporting Information Files.
2. Power calculations revealed that we would require 140 participants to detect a medium effect size (.25) with power set at .85 and *a* = .05, for a correlation bivariate normal two-tailed model; and we would require 175 participants to detect a medium effect size (.25) with power set at .85 and *a* = .05, for a chi square goodness-of-fit test.
